# Comparison of Properties of Colorless and Transparent Polyimide Nanocomposites Containing Chemically Modified Nanofillers: Functionalized-Graphene and Organoclay

**DOI:** 10.3390/polym14122469

**Published:** 2022-06-17

**Authors:** Seon Ju Lee, Moon Young Choi, Lee Ku Kwac, Hong Gun Kim, Jin-Hae Chang

**Affiliations:** 1 Graduate School of Carbon Convergence Engineering, Jeonju University, Jeonju 55069, Korea; qaz5515@naver.com (S.J.L.); ansdud2303@naver.com (M.Y.C.); kwac29@jj.ac.kr (L.K.K.); hkim1125@hanmail.net (H.G.K.); 2 Institute of Carbon Technology, Jeonju University, Jeonju 55069, Korea

**Keywords:** colorless and transparent polyimide, nanocomposite, film, poly(amic acid), thermal imidization

## Abstract

Poly(amic acid) (PAA) was synthesized from dianhydride 4,4-(4,4-isopropylidenediphenoxy)bis(phthalic anhydride) and diamine bis [4-(3-aminophenoxy) phenyl] sulfone. Colorless and transparent polyimide (CPI) hybrid films were synthesized through thermal imidization after dispersing nanofillers using an intercalation method in a PAA solution. C16-GS and C16-MMT, in which hexadecylamine (C16) was substituted on graphene sheet (GS) and montmorillonite (MMT), respectively, were used as nanofillers to reinforce the CPI hybrid films. These two nanofillers were admixed in varying loadings of 0.25 to 1.00 wt%, and the morphology, thermal properties, and optical transparency of the hybrid films were investigated and compared. The results suggest that the thermal properties of the CPI hybrid films can be improved by adding only a small amount of nanofiller. Transmission electron microscopy results of the CPI hybrid film containing two types of fillers suggested that the fillers were well dispersed in the nano-size in the matrix polymer; however, some of the fillers were observed as agglomerated particles above the critical concentration of 0.50 wt%.

## 1. Introduction

Recently, many studies have been conducted to develop highly functional polyimide (PI) materials with superior thermo-mechanical properties, excellent processability, and high optical transparency compared with conventional PI [1,2,3]. Among the newly developed PIs, colorless and transparent PIs (CPIs) have attracted significant attention in the field of electronic materials for diverse applications, such as flexible display substrates, semiconductors, and electro-optical devices [4,5,6]. Most CPIs exhibit favorable properties because the structure of the main chain is bent or comprises bulky –CF_3_ or –SO_2_ electron-withdrawing groups that prevent the formation of charge-transfer complexes (CTCs) between the chains [7,8]. Moreover, it is possible to obtain CPIs with various physical properties, depending on the design of the monomer used. In addition, these CPIs have been shown to have superior solubility, thermal stability, and high optical transparency compared with PIs that have been commercialized for several decades [9,10].

CPI can be widely applied to electronic materials and devices for display as a replacement for glass. In addition, it can be easily synthesized and mass-produced, so it can be used in solar panels, liquid crystal displays (LCD), and plasma display panels (PDP). Recently, flexible and transparent indium tin oxide (ITO) in the form of conductive oxide has been widely used in display substrates and microelectronics fields. However, since indium is rare and expensive, there are many limitations to the use of ITO glass as a display material [11]. Therefore, CPI is not only a suitable alternative to overcome the limitations of glass, but also can be used as a wearable electronic device or a transparent electrode.

Novel nanocomposite materials that contain a certain amount of nanofiller well-dispersed in an organic polymer matrix significantly improve the performance of existing materials and offer routes to material properties that cannot be achieved through traditional procedures [12,13]. Nanocomposite materials with low nanofiller loadings (<10 wt%) exhibit considerably improved thermo-mechanical properties as well as unique optical properties and morphologies owing to their excellent dispersibility. The aromatic precursor poly(amic acid) (PAA) has significant potential to emerge as a high-performance nanocomposite CPI via thermal imidization reactions with nanofillers [14,15].

Graphene and clay are well-established nanofillers. Owing to a surface area, thermal conductivity, and electrical conductivity of 2630 m^2^/g, 5000 W m/K, and 6000 S/cm, respectively, single-layer graphene harbors the excellent potential for applications, such as materials for gas barriers, transparent electrodes, and solar cells as a filler in polymeric nanocomposites [16,17,18]. However, because graphene is a two-dimensional aggregate of carbon, it is necessary to increase the compatibility thereof with the polymer matrix through chemical or physical modification for viable incorporation into a nanocomposite material and to prevent micro-phase separation. One of the efficient ways to achieve homogeneous graphene dispersion in a polymer matrix is to use functionalized-graphene sheets (F-GSs) that are obtained by separating the agglomerated carbonaceous structure through an organic reaction [19,20,21].

When clay montmorillonite (MMT) with a large surface area (700–800 m^2^/g) is used as a nanofiller in a polymer composite, the thermomechanical properties and solvent resistance of the resulting nanocomposite are significantly increased owing to the mutual attraction between the composite materials. However, because MMT is hydrophilic and therefore incompatible with lipophilic polymers, it is necessary to chemically combine MMT with a suitable organic agent. These organoclays are dispersed in the polymer as nanoparticles to effectively enhance the properties of the composite material [22,23,24].

In this study, we used 4,4’-(4,4’-isopropylidenediphenoxy)diphthalic anhydride (BPADA) as a dianhydride and bis [4-(3-aminophenoxy)phenyl] sulfone (*m*-BAPS) as a diamine to synthesize a CPI precursor, and thermal imidization was performed with dispersed nanofillers to synthesize CPI hybrid films. BPADA is commonly used to synthesize CPI and is known to improve solubility and colorless transparency. To prevent CTC formation and increase the optical transparency, BPADA and *m*-BAPS were used to fabricate an overall curved CPI structure. The F-GS (C16-GS) and organoclay (C16-MMT) nanofillers were synthesized by reacting hexadecylamine (C16) with GS and MMT, respectively. The same organic agent was used to enable contrasting effects of GS and MMT on the CPI hybrid films. Filler loadings of 0.25 to 1.00 wt% with respect to the polymer matrix were investigated.

The purpose of this study is to evaluate the dispersion effect according to the two nanofillers used in the CPI hybrid and the various contents. A method to manufacture a CPI hybrid film through solution intercalation is described, and thermal properties, optical transparency, and morphologies of the CPI hybrid films are explored. 

## 2. Materials and Methods

### 2.1. Materials

BPADA and *m*-BAPS were purchased from TCI (Tokyo, Japan). Hexadecylamine and *N*,*N*’-dimethylacetamide (DMAc) from Aldrich (Yongin, Korea). DMAc was dried on a molecular sieve (4 Å). Graphene oxide (GO) was purchased from Standard Graphene Co. (Ulsan, Korea). Na^+^-MMT was obtained from Aldrich (Yongin, Korea), and the cation exchange capacity was 119 meq/100 g. 

### 2.2. Syntheses of Nanofillers

#### 2.2.1. Synthesis of C16-GS

A mixture of 0.5 g GO dispersed in 1.5 L H_2_O and 1.0 g C16 dissolved in 25 mL EtOH was allowed to react at room temperature in a nitrogen atmosphere for more than 12 h. The mixture was filtered at a reduced pressure to obtain a powder, which was washed using 1:1 H_2_O:EtOH (*v*:*v*) and dried under vacuum at 70 °C for 24 h [25]. A black powder was obtained in approximately 90% yield. 

#### 2.2.2. Synthesis of C16-MMT

C16 (11.47 g) was added to a solution of 100 mL H_2_O and 4.8 mL HCl in a nitrogen atmosphere at 80 °C for 1 h. In a separate beaker, 20 g Na^+^-MMT was dispersed in 400 mL H_2_O at 80 °C for 1 h. The two solutions were subsequently mixed and stirred vigorously at 80 °C for 2 h. A white powder obtained via filtration at reduced pressure was washed using 1:1 distilled water:ethanol (*v*:*v*) and freeze-dried under vacuum at room temperature for 24 h [26]. The yield of the white powder obtained was approximately 95%.

### 2.3. Synthesis of PAA

The purity, molar ratio, concentration, and solvent of PAA are important factors in determining the properties of CPI. After dissolving 6.77 g (1.3 × 10^−2^ mol) BPADA in 40 mL DMAc under nitrogen, the mixture was stirred at 25 °C for 30 min. A solution of 5.62 g (1.3 × 10^−2^ mol) of *m*-BAPS in 40 mL DMAc was mixed with the BPADA solution and stirred at room temperature for 16 h.

### 2.4. Preparation of the CPI Hybrid Film

**Scheme 1:**
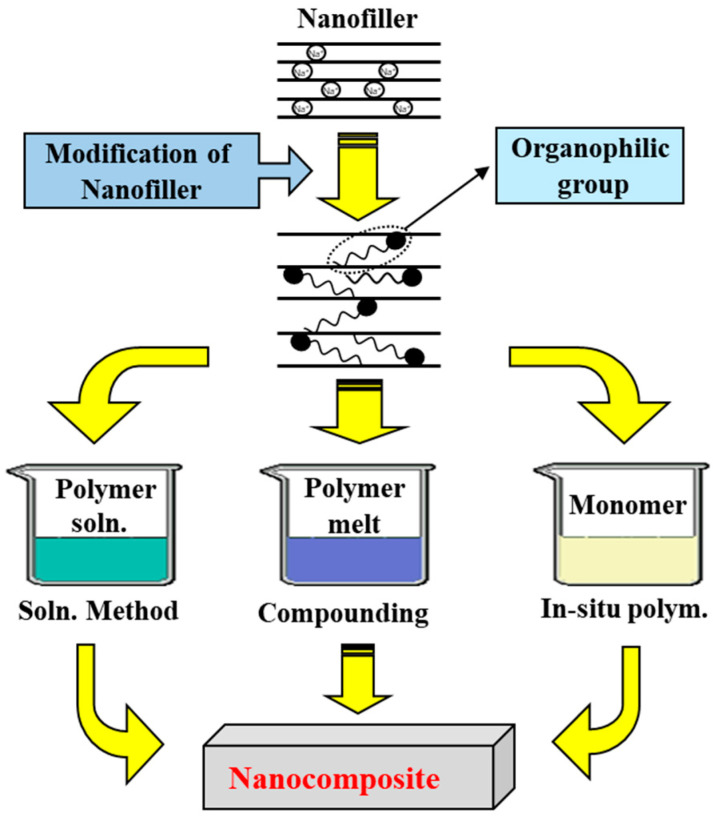
Several methods for preparing nanocomposites.

There are three major methods for preparing a nanocomposite using a filler: a solution intercalation method, a melt intercalation method, and an in situ polymerization method [27]. Among them, the solution intercalation method requires that the filler and the polymer have compatibility and be well dispersed or dissolved at the same time in the specific solvent used. From this point of view, it is difficult to select a solvent, and post-treatment of the solvent after production is difficult. However, the solution intercalation method is mainly used in the process using a solvent [28]. In this study, the solution intercalation method was used because the monomers for PI synthesis were reacted in DMAc solvent. Several methods for preparing nanocomposites are shown in Scheme 1.

**Scheme 2:**
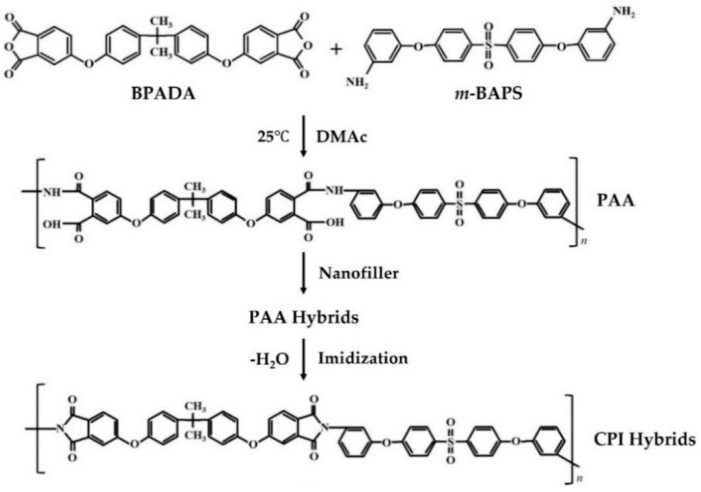
Synthesis route for the fabrication of CPI hybrid films.

The same procedure was followed to produce all CPI hybrid films; therefore, the synthesis procedure of only a CPI hybrid film containing 0.5 wt% C16-GS is described as a representative example. A mixture of 0.06 g C16-GS in 70 mL DMAc was dispersed via sonication at room temperature for 3 h. The solution was then added to the prepared PAA solution and sonicated for 1.5 h. The PAA hybrid solution was cast onto a glass plate and placed under vacuum at 50 °C for 2 h and at 80 °C for 1 h to remove the solvent. Thereafter, CPI hybrid films were prepared via thermal imidization at various temperatures and durations. The reaction conditions are listed in Table 1, and the overall chemical structure of the materials and reaction steps for the CPI hybrid synthesis are shown in Scheme 2. Nanofiller loadings were varied from 0 to 1.0 wt%.

### 2.5. Characterization

#### 2.5.1. Atomic Force Microscopy (AFM), Field Emission Scanning Electron Microscopy (FE-SEM), and Transmission Electron Microscopy (TEM) Analyses

An AutoProbe CP/MT scanning probe microscope was used to obtain images during AFM analysis (Multimode, NanoScope III, Digital Instruments Inc., New York NY, USA). After dispersing the samples in a solvent, the solution was sonicated for 3 h and spin-coated on a silicon wafer.

To observe the cross-section of a film, the sample was quenched in liquid nitrogen, segmented, and then examined using FE-SEM (JEOL, JSM-6500F, Tokyo, Japan). To increase the conductivity of the segmented surface, it was sputter-coated with gold using an SPI sputter coater. TEM (JEOL, JEM 2100, Tokyo, Japan) was used to investigate the morphology of graphene and clay dispersed in the CPI matrix. The specimens were cured with epoxy resin at 70 °C for 24 h, and samples with a thickness of 90 nm were prepared under vacuum using a microtome equipped with a glass knife. The TEM acceleration voltage was 120 kV.

#### 2.5.2. Fourier Transform Infrared (FT-IR) and ^13^C Magic Angle Spinning-Nuclear Magnetic Resonance (MAS NMR) Spectroscopy Analyses

FT-IR spectroscopy (PerkinElmer, Spectrum Two, Llantrisant, UK) was used to confirm the synthesis of the nanofillers and CPI. Room-temperature solid-state ^13^C cross-polarized (CP)/MAS NMR spectroscopy (Bruker 400 DSX NMR, Berlin, Germany) was performed at a Larmor frequency condition of 100.61 MHz. Chemical shifts were corrected using tetramethylsilane as reference.

#### 2.5.3. X-ray Diffraction (XRD) Analysis

A wide-angle X-ray diffractometer (Rigaku, SWXD/X-MAX/2000PC, Tokyo, Japan) was used to determine the interlayer distance of the nanofiller dispersed in the CPI matrix using Cu-Kα (*λ* = 1.5406 Å) radiation. The measurement range was 2*θ* = 2°–15° at a scan speed of 2°/min. 

#### 2.5.4. Thermal Analysis

Differential scanning calorimetry (DSC, 2-00915, Delaware, USA) and thermogravimetric analysis (TGA, SDT 0650-0439, Delaware, USA) were used to evaluate the thermal properties of the CPI film, and the temperature increase during measurement was 20 °C/min. Thermomechanical analysis (TMA, SS6100, Tokyo, Japan) was employed for measuring the coefficient of thermal expansion (CTE) using a sample with dimensions of 5 × 30 mm^2^, and the heating rate was 5 °C/min under the condition of an expansion force of 0.1 N. The resulting values were obtained via secondary heating.

#### 2.5.5. Optical Properties

The cutoff wavelength (*λ_o_*) and transmittance between 300 and 800 nm of 53–58 μm-thick films were measured using an ultraviolet-visible (UV-vis) spectrometer (SHIMADZU UV-3600, Tokyo, Japan). The yellow index (YI) of the CPI and CPI hybrid films was measured using a spectrophotometer (KONICA MINOLTA CM-3600D, Tokyo, Japan).

## 3. Results and Discussion

### 3.1. Nanofillers

**Figure 1:**
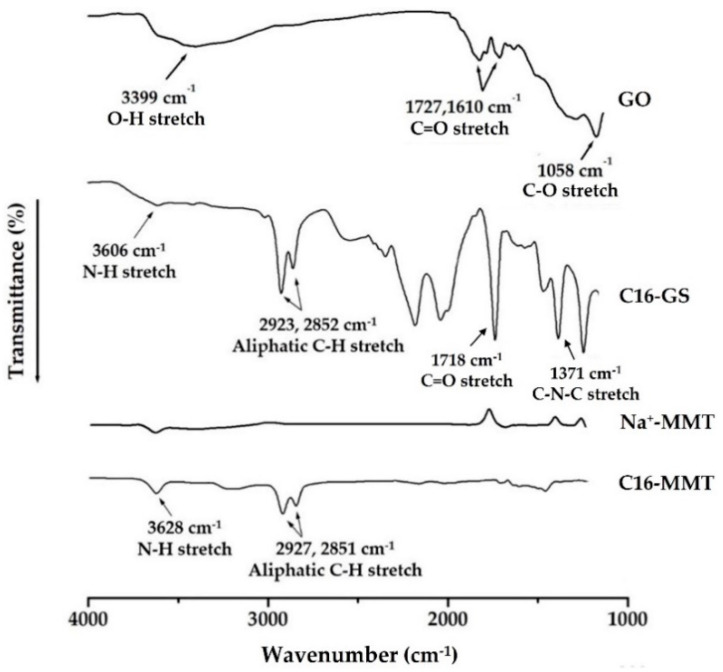
FT-IR spectra of GO, F-GS (C16-GS), Na^+^-MMT, and organoclay (C16-MMT).

Figure 1 shows the FT-IR spectra of the starting materials GO and MMT as well as their C16 chemically substituted derivatives C16-GS and C16-MMT. In the GO spectrum, peaks are observed corresponding to O–H (3399 cm^−1^), C=O (1727 and 1610 cm^−1^), and C–O (1058 cm^−1^) stretching. After reacting GO with C16 (C16-GS), peaks related to N–H stretching, aliphatic C–H stretching, and C=O stretching are observed at 3606, 2923 and 2852, and 1718 cm^−1^, respectively. C-N-C stretching peak was also observed at 1371 cm^−1^. Although no peaks were observed in the spectrum of pure MMT, N–H (3628 cm^−1^), and aliphatic C–H (2927 and 2851 cm^−1^) stretching peaks were observed in the spectrum of the organoclay C16-MMT [29].

**Figure 2:**
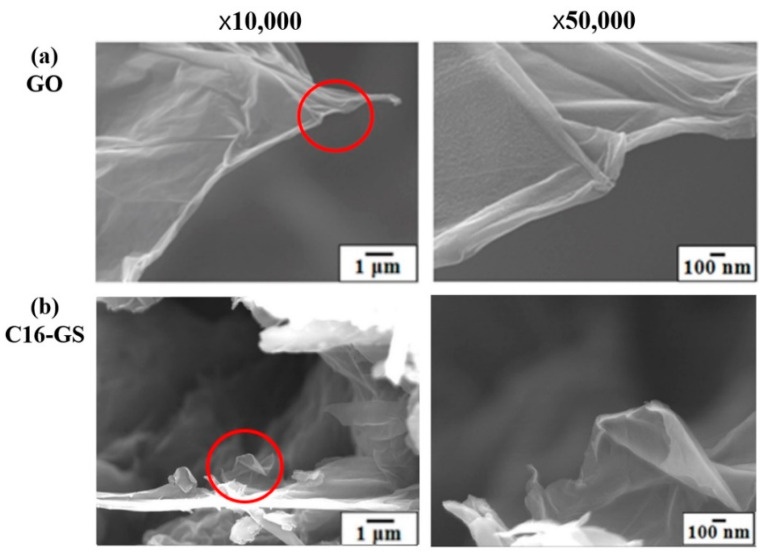
FE-SEM photographs of (**a**) GO and (**b**) C16-GS.

SEM images of GO and C16-GS are shown in Figure 2. GO exhibited sheets that remain translucent even when bent and folded and were well-separated from the graphite surface (Figure 2a). C16-GS was transparent and had a thin, wrinkled morphology because the substitution with C16 caused significant damage to the graphene surface (Figure 2b). In general, F-GS substituted with a long alkyl group exhibited higher porosity and volume expansion compared to GO and had a very low bulk density. Therefore, melt compounding using F-GS presents significant processing challenges, and a masterbatch is usually required to mitigate this issue [18]. However, these processing difficulties can be overcome by mixing the polymer and F-GS in the solution phase.

**Figure 3:**
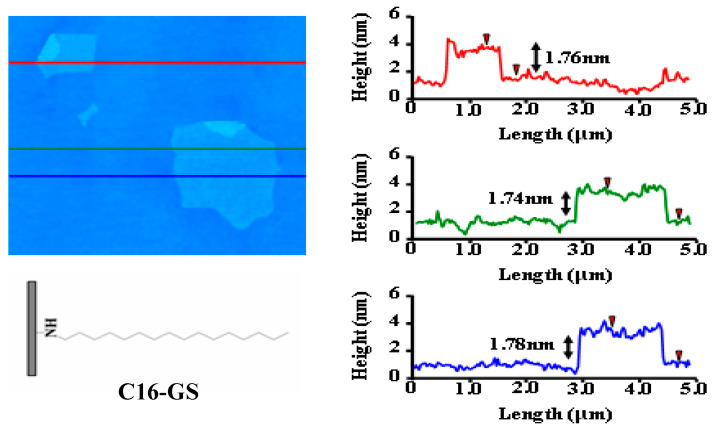
Non-contact mode AFM image of C16-GS with three height profiles acquired at different locations.

Graphene is composed of individual carbon layers with a thickness of approximately 1 nm. However, graphene most often agglomerates or bundles to form several layers, resulting in significant differences between the theoretical and true thickness values. AFM can provide information regarding the surface topology, defects, dimensions, thickness, and bending properties of GSs. For monolayer graphene and F-GS, the lateral size and thickness can be measured using stepped height scans. An AFM image of C16-GS deposited as a dispersion on a mica substrate is shown in Figure 3, indicating that the average thickness of the graphene layers is 1.76 nm. We previously reported that the interlayer spacing of graphene layers increases as the length of the alkyl group in F-GS increases [30]. 

### 3.2. CPI Structure

**Figure 4:**
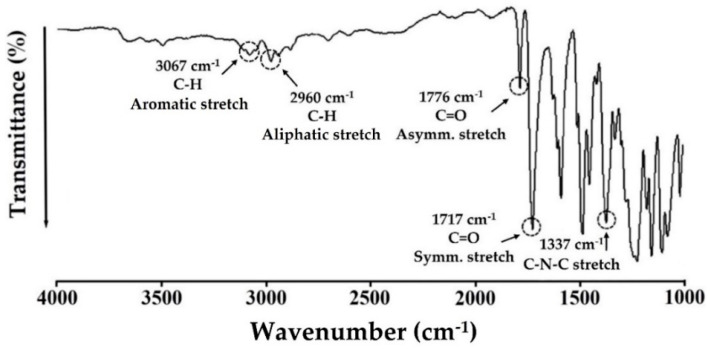
FT-IR spectra of CPI.

**Figure 5:**
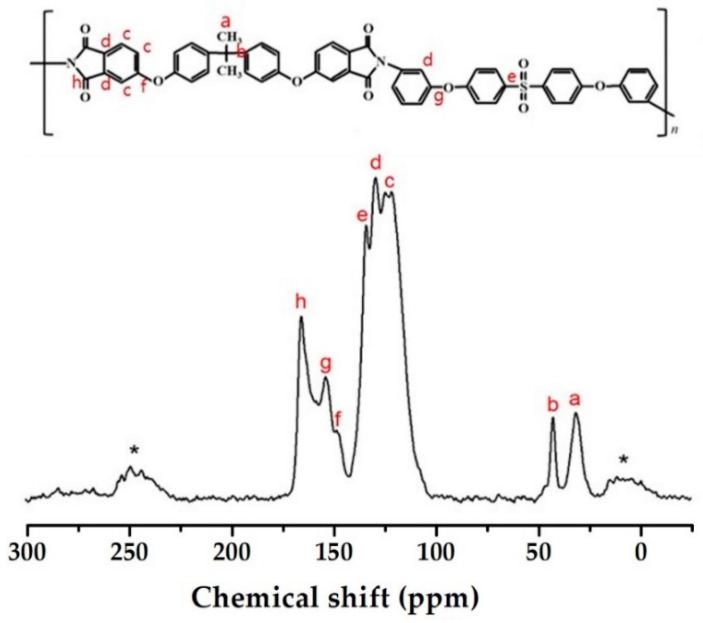
^13^C MAS NMR spectra of CPI.

The synthesis of CPI was confirmed using FT-IR and ^13^C MAS NMR spectroscopy, and the results are shown in Figure 4 and Figure 5, respectively. Peaks corresponding to aromatic and aliphatic C–H stretching were observed at 3065 and 2960 cm^−1^, respectively, and peaks corresponding to –C=O stretching were detected at 1776 and 1717 cm^−1^ (Figure 4). In addition, the imide C–N–C stretching at 1337 cm^−1^ confirmed successful imidization [29]. NMR analysis revealed peaks corresponding to methyl (–CH_3_), isopropyl (–C–(CH_3_)_2_), and carbonyl (–C=O) groups of the PI structure at 30.96, 42.42, and 165.26 ppm, respectively (Figure 5). Phenyl carbon peaks were observed at 127.25, 133.01, 142.67, and 151.25 ppm with spinning sidebands (indicated by an asterisk) [29]. 

### 3.3. XRD Analysis

**Figure 6:**
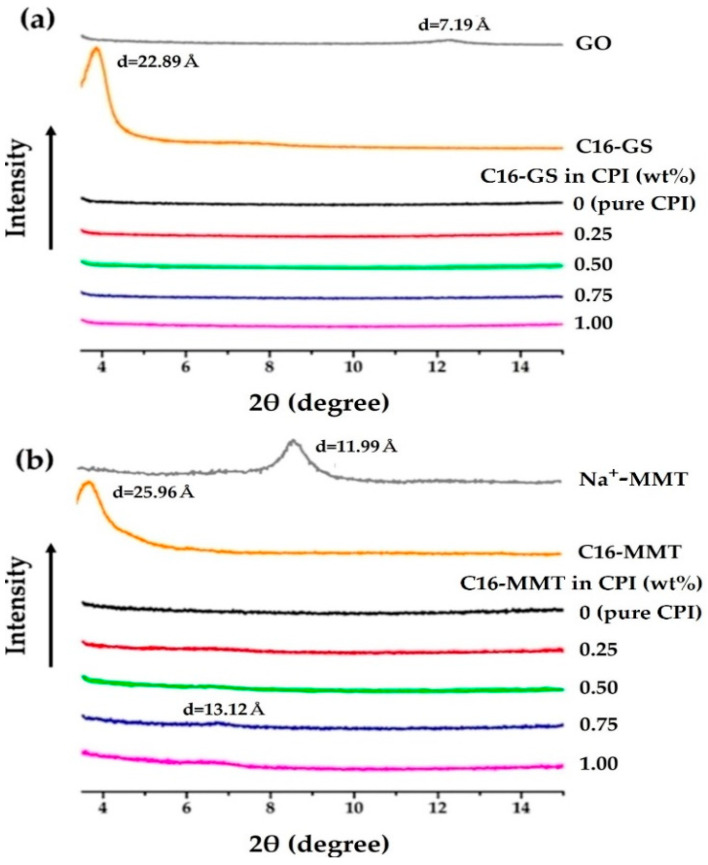
XRD patterns of nanofillers, CPI, and CPI hybrid films with various loadings of nanofillers (**a**) C16-GS and (**b**) C16-MMT.

XRD patterns in the range 2*θ* = 3°–15° of GO, C16-GS, MMT, C16-MMT, and CPI hybrid films with varying nanofiller loadings are shown in Figure 6. GO exhibited a weak peak at 2*θ* = 12.30°, while C16-GS showed a very sharp peak at 2*θ* = 3.86° (Figure 6a). The interlayer spacing *d* of C16-GS (*d* = 22.89 Å) was larger than that of GO (*d* = 7.19 Å) owing to the long alkyl group substituent on the GO surface. For the CPI hybrid film, no F-GS peak was observed until a loading of 1.00 wt%. This indicates that C16-GS is completely dispersed in the CPI matrix with no agglomeration.

A diffraction peak was observed at 2*θ* = 8.60° (*d* = 11.99 Å) for pristine MMT, while C16-MMT exhibited a peak at 2*θ* = 3.69° (*d* = 25.96 Å) (see Figure 6b). The significant increase in the interlayer spacing of the organoclay was ascribed to the substitution of the clay surface with the long alkyl chain [26,31]. No clay peak was observed up to a 0.50 wt% C16-MMT loading; however, at a loading of 0.75 wt%, a very small peak appeared at 2*θ* = 6.73° (*d* = 13.12 Å), and the intensity of this peak increases for a loading of 1.00 wt%. This observation can be explained by the agglomeration of the organoclay nanofiller above a certain critical concentration [32,33,34].

Because the interlayer separation of C16-MMT (*d* = 25.96 Å) was larger than that of C16-GS (*d* = 22.89 Å), it is easier to insert a polymer chain when C16-MMT is used as the nanofiller rather than C16-GS, and thus, improved hybrid properties can be expected [22,26,31].

In general, XRD analysis is used to investigate the aggregation and interlayer separation of the composite. However, it provides only a preliminary indication, and it is necessary to observe the insertion or exfoliation of nanofillers using electron microscopy [35,36].

### 3.4. Morphology

**Figure 7:**
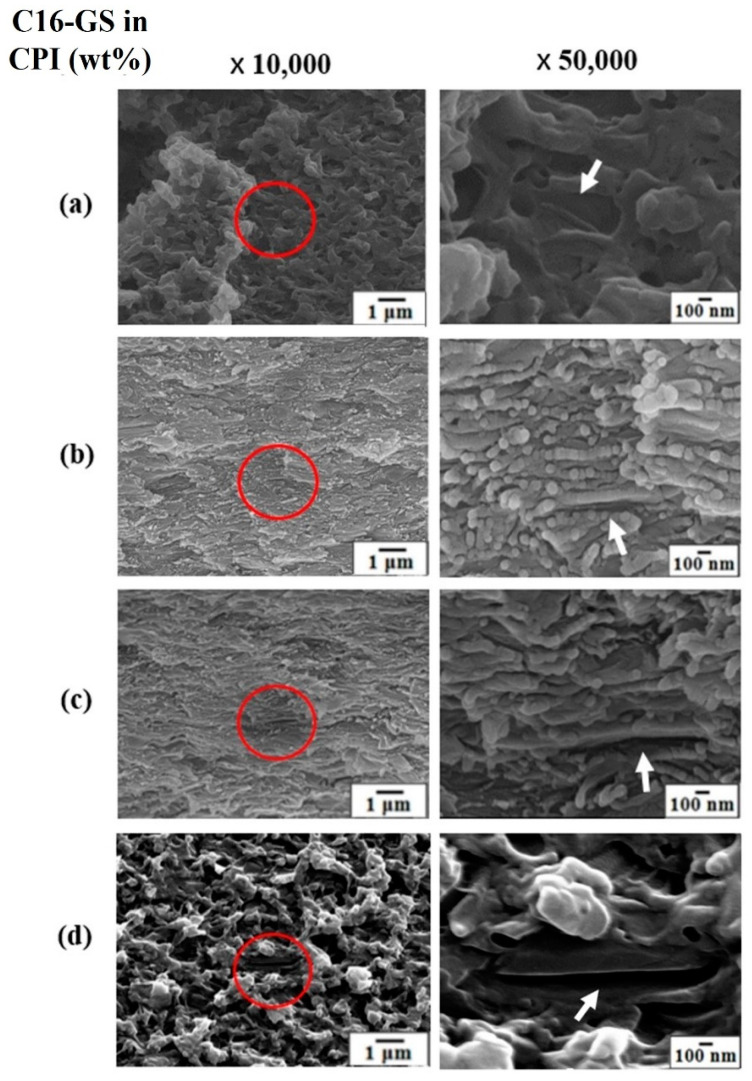
FE-SEM micrographs of CPI hybrid films containing (**a**) 0.25, (**b**) 0.50, (**c**) 0.75, and (**d**) 1.00 wt% C16-GS.

FE-SEM was used to observe the fracture surfaces of CPI hybrid films with various C16-GS loadings, and micrographs are shown in Figure 7. The GSs are distributed in a uniform direction in the form of straight plates. Following initially good dispersibility, the agglomeration of F-GS in the CPI matrix gradually increases with increasing loading. These results show that as the amount of C16-GS increased, several empty spaces were detected in the matrix, indicating poor dispersibility in the CPI matrix. From the results of this study, the dispersibility of F-GS increased up to a certain concentration but decreased above the critical concentration.

**Figure 8:**
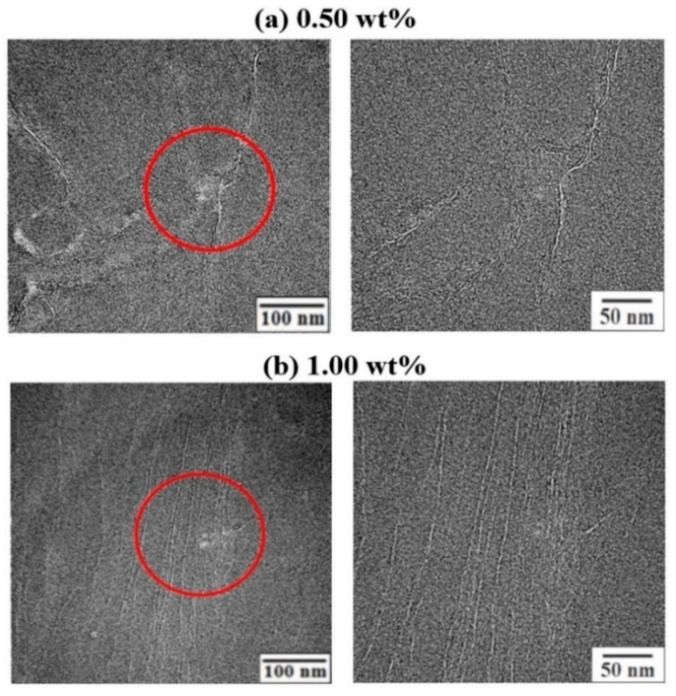
TEM micrographs of CPI hybrid films containing (**a**) 0.50 and (**b**) 1.00 wt% C16-GS with different magnifications.

TEM was used to confirm the dispersed morphology of C16-GS in the CPI polymer matrix. Figure 8 shows TEM images of the hybrid films containing 0.50 and 1.00 wt% C16-GS. The bright white line represents the graphene layer, and the space between the white lines corresponds to the CPI matrix. In the film containing 0.50 wt% C16-GS (Figure 8a), the GSs were exfoliated and randomly dispersed over a large area. However, at 1.00 wt% loading (Figure 8b), agglomerated GSs with a uniform arrangement were observed. This observation agrees well with the FE-SEM results, and the negative effect of agglomeration on the thermal and optical properties is described below. 

**Figure 9:**
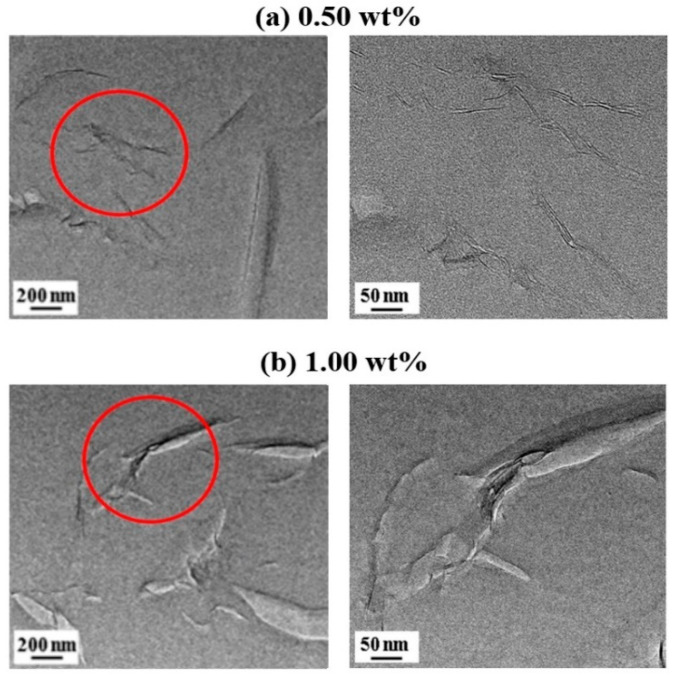
TEM micrographs of CPI hybrid films containing (**a**) 0.50 and (**b**) 1.00 wt% C16-MMT with different magnifications.

Organoclay is well-dispersed in the CPI polymer matrix for a loading of 0.50 wt% (Figure 9a). However, at a C16-MMT loading of 1.00 wt%, the clay showed an overall agglomerated morphology (Figure 9b), which corroborates the findings from the XRD analysis (Figure 6b). C16-GS is uniformly oriented and has a straight and rigid plate shape, whereas C16-MMT is randomly oriented and well-dispersed in the matrix, which improves the physical properties of the hybrid film [37].

### 3.5. Thermal Properties

**Figure 10:**
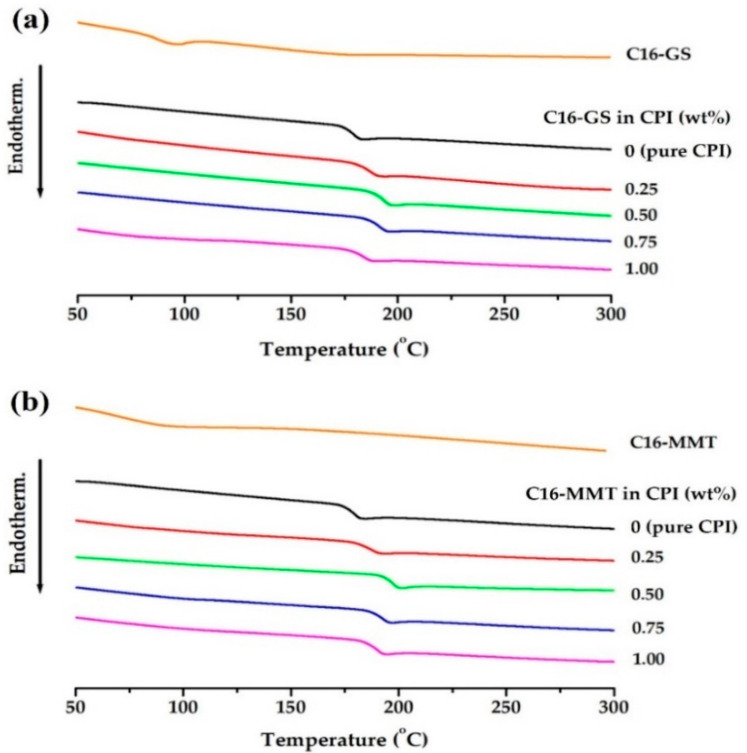
DSC thermograms of nanofillers, CPI, and CPI hybrid films with various nanofiller loadings of (**a**) C16-GS and (**b**) C16-MMT.

Figure 10 shows the DSC thermograms of the CPI hybrid films with various nanofillers loadings, and Table 2 summarizes their thermal properties. The glass transition temperature (*T_g_*) of the pure CPI film is 170 °C and that of the hybrid film gradually increases with the nanofiller content. For example, the *T_g_* values increased to 185 and 191 °C for 0.50 wt% C16-GS and C16-MMT, respectively. This reflects the decreased mobility of the polymer chains sandwiched between hard plate-shaped nanofiller layers. Eventually, the segmental motion of the polymer chain was disturbed, and *T_g_* increased. The increase in *T_g_* is explained by two factors [38,39]. First, the nanofiller layers dispersed in the polymer matrix significantly decrease the free volume of polymer chains, thus increasing the *T_g_* of the hybrid. The second factor is the constraint of the polymer chains inserted inside the filler gallery, which prevents the segmental motion of the polymer chains.

However, when the content of the nanofillers increased from 0.50 to 1.00 wt%, the *T_g_* value of the hybrid decreased to 177 and 183 °C for C16-GS and C16-MMT, respectively. This is ascribed to filler agglomeration, which occurs when nanofiller loading exceeds a critical concentration. Similar results have been reported in several papers [40,41]. 

CPI hybrid films comprising C16-MMT possess higher *T_g_* values than films containing C16-GS. This is because the aspect ratio of MMT (~218) is smaller than that of GS (>250), which facilitates the dispersion of MMT in the matrix polymer [18,23]. This result confirms the dispersion state, as indicated by the electron microscopy results (Figure 7, Figure 8 and Figure 9).

**Figure 11:**
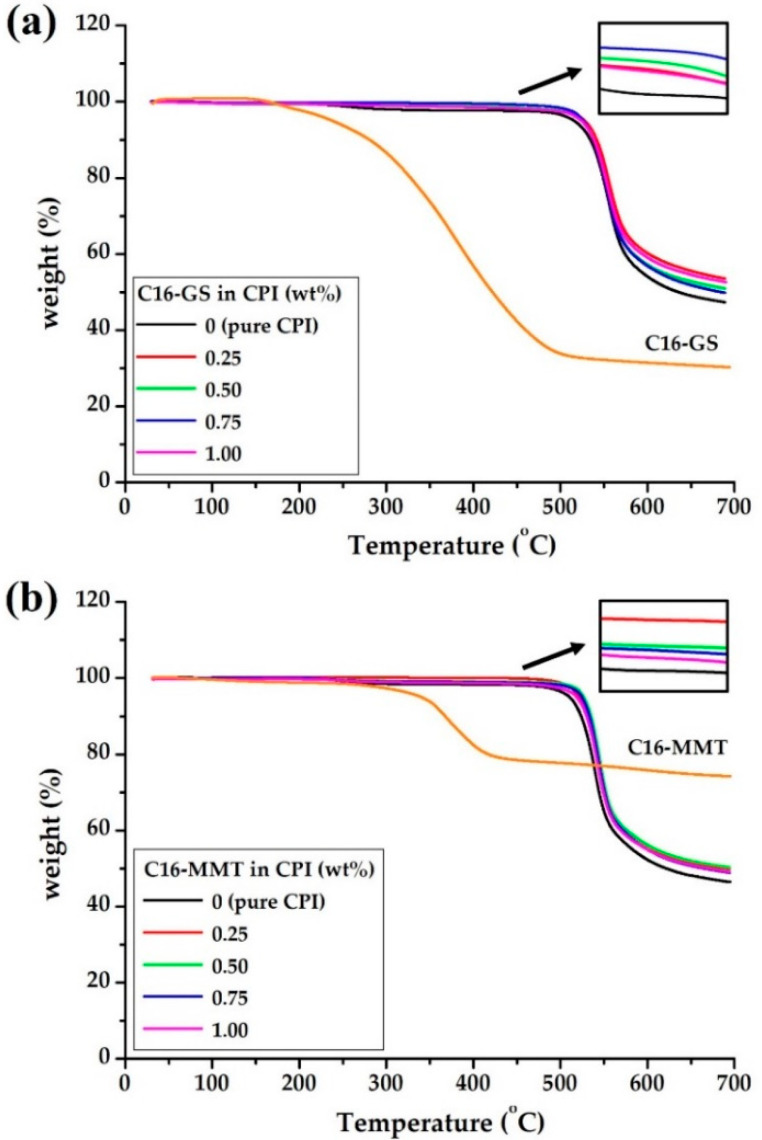
TGA thermograms of nanofillers, CPI, and CPI hybrid films with various nanofiller loadings of (**a**) C16-GS and (**b**) C16-MMT.

From the TGA results (Figure 11), the initial 2% weight loss temperature (*T_D_^i^*) of the pure CPI film was 467 °C (Table 2), and the 0.50 wt% C16-GS hybrid has a *T_D_^i^* value of 492 °C. This was rationalized by dispersed C16-GS hindering heat transfer, thereby suppressing volatilization [42,43,44]. However, increasing the C16-GS loading from 0.50 to 1.00 wt% results in a lower *T_D_^i^* value of 467 °C, which is ascribed to nanofiller agglomeration. The C16-MMT hybrid film exhibited the same trend, attaining a maximum *T_D_^i^* value of 511 °C for loading of 0.50 wt%, and as the nanofiller loading was increased to 1.00 wt%, the *T_D_^i^* value decreased to 483 °C. 

As shown in Figure 11, all the thermograms of the hybrid films exhibit a small weight loss at ~250 °C, owing to the decomposition of C16, and the loss starting at about 450 °C is presumed to be the decomposition of the PI main chain.

The weight residue at 600 °C (*wt_R_*^600^) of the pure CPI film did not significantly change with the admixture of either nanofiller. This result is due to the retention of the excellent heat resistance of the plate-like layers of GS and MMT after the decomposition of the substituted alkyl groups. 

Upon heating the polymer, the parallel PI chains relax in a direction perpendicular to their propagation direction. The GS or MMT layers of the nanofillers are considerably harder to deform. Therefore, graphene or clay, which can efficiently block heat transfer, effectively inhibits the traverse thermal expansion of the polymer matrix [45,46,47]. To endow hybrid films with high resistance to thermal expansion, the matrix polymer and nanofiller must have high thermal stability. 

**Figure 12:**
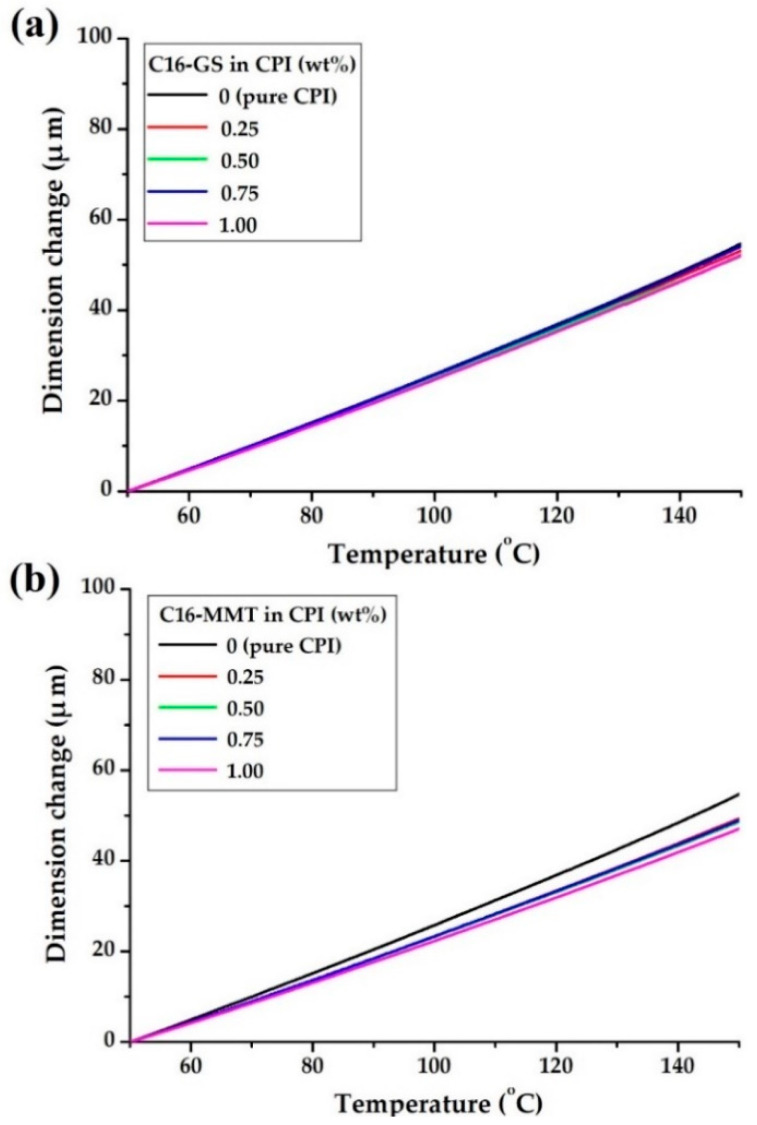
TMA thermograms of nanofillers, CPI, and CPI hybrid films with various nanofiller loadings of (**a**) C16-GS and (**b**) C16-MMT.

Table 2 summarizes the CTE values of the CPI hybrid films with various nanofillers loadings obtained after secondary heating in the temperature range of 50–150 °C. In the C16-GS hybrid, CTE values were approximately constant in the range of 52.0–54.7 ppm/°C regardless of the nanofiller content. However, the CTE value decreases with C16-MMT loading. For example, the CTE value decreased from 54.7 ppm/°C for pure CPI to 47.1 ppm/°C for the 1.00 wt% C16-MMT hybrid film. Figure 12 shows the TMA thermograms of the CPI hybrid films with different nanofiller loadings.

Overall, the thermal properties of the C16-MMT hybrid film were superior to those of the C16-GS hybrid film. This is attributed to the following reasons. (1) C16-MMT had a larger *d* than C16-GS, which facilitates the intercalation of polymer chains (Figure 6). (2) MMT has a shorter aspect ratio than GS, which facilitates dispersion in a polymer matrix. (3) The thermal stability of the organoclay was higher than that of F-GS. According to the TGA results (Figure 11 and Table 1), the *T_D_^i^* of C16-GS and C16-MMT were 196 and 279 °C, respectively. Owing to the superior thermal stability of C16-MMT compared to C16-GS, the thermal stability (*T_D_^i^*, *wt_R_*^600^, and CTE) of the hybrid using the organoclay nanofiller is also superior to that of the F-GS hybrid. 

### 3.6. Optical Properties

**Figure 13:**
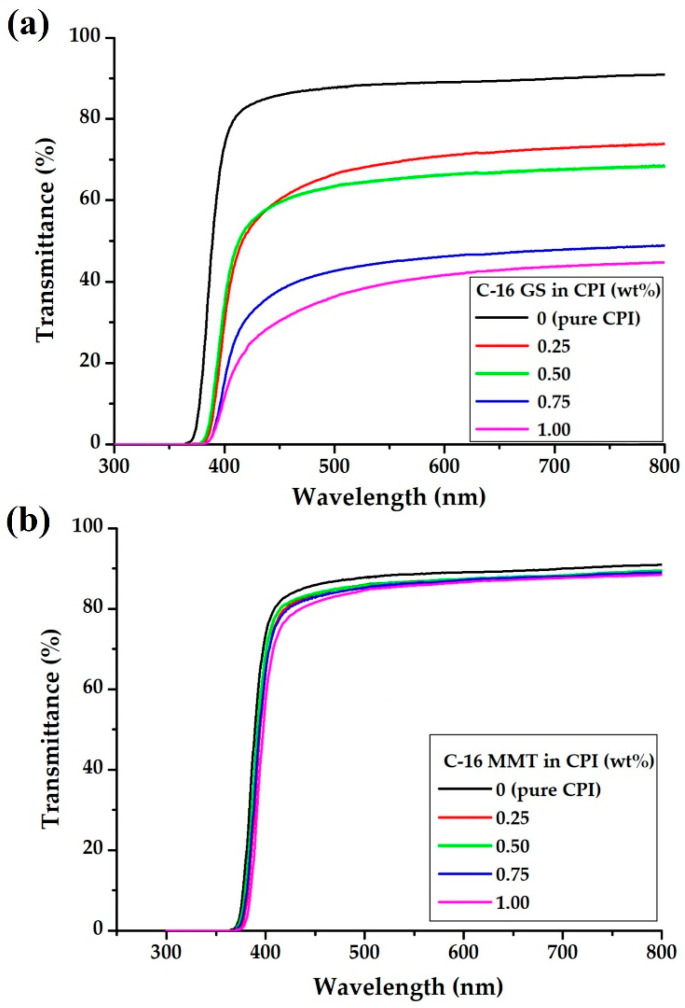
UV-vis transmittance of nanofillers, CPI, and CPI hybrid films with various nanofiller loadings of (**a**) C16-GS and (**b**) C16-MMT.

Figure 13 shows the UV-vis transmittance spectra of the hybrid films with various nanofiller loadings, and the *λ_o_* and transmittance at 500 nm (500 nm^trans^) values are listed in Table 3. The *λ_o_* value of pure CPI (368 nm) gradually increased with the content of the two nanofillers. For example, the *λ_o_* value of the 0.25 and 1.00 wt% C16-GS hybrid films are 380 and 385 nm, respectively, and that of the 1.00 wt%C16-MMT hybrid film is 377 nm. All the hybrid films obtained in this study exhibit *λ_o_* values below the visible light wavelengths (400–800 nm).

The 500 nm^trans^ value substantially decreases from 87% to 36% when the C16-GS loading increased from 0 to 1.00 wt%; however, it remained constant regardless of the organoclay loading (Table 3). Similarly, the YI value of the film significantly increases from 2 to 15 when the C16-GS content increases to 1.00 wt%, while it was maintained in the range of 1–3 for all C16-MMT loadings. Therefore, colorlessness and transparency were maintained for the organoclay nanofiller.

The optical transparencies of the C16-MMT hybrid film with the same filler contents were better than those of the C16-GS hybrid film. As observed from the TEM images in Figure 8 and Figure 9, at the same loading, C16-MMT had a smaller plate shape and better dispersion than C16-GS; therefore, the optical properties of pure CPI were better maintained and remained largely unaffected by the organoclay content. For C16-GS hybrid films, the transmittance decreased, and the YI value increased as the GS content increased because the plate aspect ratio was larger than that of MMT, and the dispersion state was worse than that of MMT. As already explained, these results are closely related to the aspect ratios of the GS and MMT.

## 4. Conclusions

In this study, we aimed to examine the effect of different types of nanofillers on the photophysical properties of the CPI matrix. F-GS and organoclay nanofillers were obtained by chemically substituting C16 alkyl groups onto GO and Na^+^-MMT, respectively. CPI hybrid films were prepared by dispersing F-GS and organoclay as nanofillers in the CPI matrix at loadings of 0.25 to 1.00 wt%. C16-MMT has a smaller plate size and better dispersion than C16-GS in CPI. The thermal properties of both types of hybrid films were optimal at 0.50 wt% loadings. Although slightly poorer than that of the pure CPI film, the optical transparency of the C16-MMT hybrid film was significantly better than that of the C16-GS hybrid films.

CPI has been widely used in applications requiring high-performance plastics because of its excellent thermo-mechanical properties and optical transparency. These excellent physical properties and applicability can be extended by appropriately controlling the structure of the monomers constituting CPI composites. The high interfacial adhesion resulting from the uniform dispersion of nanofillers in the CPI matrix confers nanocomposites with excellent physical properties that cannot be obtained through conventional manufacturing processes. These enhanced nanocomposites can be used as film materials in electronic and optical applications, which are currently being extensively investigated.

## Figures and Tables

**Table 1:** Heat treatment conditions of the CPI hybrid film.

Sample	Temperature (°C)/Time (h)/Pressure (torr)
PAA	25/16/760 → 50/2/1 → 80/1/1
CPI Hybrid	110/0.5/1 → 140/0.5/1 → 170/0.5/1 → 195/0.8/1 → 220/0.8/1 → 235/2/1

**Table 2:** Thermal properties of CPI hybrid films.

Filler in CPI (wt%)	C16-GS				C16-MMT			
	*T_g_* (°C)	*T_D_^i^*^a^ (°C)	*wt_R_*^600^^b^ (%)	CTE ^c^ (ppm/°C)	*T_g_* (°C)	*T_D_^i^* (°C)	*wt_R_*^600^ (%)	CTE (ppm/°C)
0(pure CPI)	170	467	52	54.7	170	467	52	54.7
0.25	180	474	54	53.2	180	508	56	49.3
0.50	185	492	55	52.0	191	511	56	49.0
0.75	181	480	52	54.2	185	504	55	49.0
1.00	177	467	54	52.2	183	483	55	47.1

^a^ Initial 2% weight loss temperature. ^b^ Weight residue at 600 °C. ^c^ Coefficient of thermal expansion for 2nd heating between 50–150 °C.

**Table 3:** Optical properties of CPI hybrid films.

Filler in CPI (wt%)	C16-GS				C16-MMT			
	Thickness (μm)	*λ_o_*^a^ (nm)	500 nm^trans^ (%)	YI ^b^	Thickness (μm)	*λ_o_* (nm)	500 nm^trans^ (%)	YI
0(pure CPI)	54	368	87	2	54	368	87	2
0.25	55	380	66	10	55	368	86	1
0.50	55	380	63	9	54	370	86	2
0.75	58	383	43	9	53	372	85	2
1.00	57	385	36	15	54	377	85	3

^a^ Cut-off wavelength. ^b^ Yellow index.

## Data Availability

The data presented in this study are available on request from the corresponding authors.
